# Observations on the Healthy and Diseased Properties of the Blood

**Published:** 1832-10-01

**Authors:** 


**Observations on the Healthy and Diseased Properties of the Blood.** By *William Stevens*, M.D. Octavo, pp. 504. Murray, London, 1831.

This is the most trumpery book, for its size, which it has ever fallen to our lot to review. Not but what it is prettily got up, clearly printed, cleanly pressed, accurately folded ; manufactured, in short, in a style not disgraceful to Mr. Murray, that Leviathan of booksellers. The printer has done his part—the only fault is in the author. And, with all the intelligence of this century, can no means be found of converting twaddle into logic—false facts into true ones? can no solvent be discovered, which shall fuse the crude fæces of weak men's brains into some precious and goodly stuff? Alas, no! nonsense, with all its trappings, is nonsense still; and the art of all the houses of Paternoster Row and Albemarle Street cannot do more than dress the doll; cannot give resistance and durability to their thing of wax.

Some persons' minds are most felicitously constituted; they believe that they are born to enlighten mankind, and, Shiloh-like, they look upon their stripes as the evidence of their mission. Happy fools! propound your schemes, concoct your theories—if the world laughs at you, laugh at it—if it beats you, defy it—if it for a moment credits you, gull it. The persons we have addressed are not the offspring of this age only, but have certainly adorned every century that has elapsed since man lost Paradise, and fools had the world before them;

——————— In vain decried and curst,
Still Dunce the second reigns like Dunce the first.

At the present day the race is undoubtedly numerous; and the mighty power of the press has tended to multiply them, as Hamlet tells us that the sun breeds maggots. It is often the reviewer's business to encounter these creatures, and it may be supposed that the task is a loathsome one. Who would not rather hunt the boar than the bug? Dulness, unopposed, is more dangerous to science than great, but intellectual error; for one debases and emasculates the mind, whilst the other only tyrannizes over it.

It is well for the human race that the numerous spawn of Dulness are short-lived; generated with facility and dispatch, their existence is little more than ephemeral. But, if the power of the press is wielded with intrepidity and talent, if men of education and of honesty direct it, the sons of Dulness may be strangled in their birth, and the small amount of mischief they are capable of inflicting on the best energies of the species is rendered smaller still.

Our readers will perceive, as we go on, whether these observations can in any way be applicable to the work before us. Dr. Stevens dedicates his production to the King of Denmark, and we cannot but admire the transcendent style in which he sets forth the transcendent virtues of that Scandinavian potentate.* The following passage is rather too difficult of digestion, with all the seasoning of Dr. Stevens' salts:—"The heartfelt interest which you are *known* to take in whatever concerns the welfare of *the human race*, has secured respect and esteem to your Majesty *in every civilized country*." This is pretty well for a beginning, and must create a pleasing anticipation of the candour and judgment of the curer of yellow fever and cholera. Yes, of yellow fever *and* cholera; and that by the self-same slops. When a footman gets his patent for a paste, it is shewn to possess the power of curing all diseases of the rectum, however they may differ from each other. This is the orthodox course of quackery. When a weak enthusiast imagines that he has found a specific for one malady, he is gradually led

* The King of Denmark takes the title of King of the Goths and Vandals; Dr. S. seems to chuckle mightily at having been a loyal subject of this King in his West India Islands—a Vandal of the New World.

from step to step, till at last he recommends his drug in all cases, and firmly believes that its powers are real. This is the ordinary course of folly. Let us see what Dr. Stevens has done. He came to England to tell his countrymen that at last yellow fever had lost its terrors, and was not only curable but had been cured by salts. No sooner did cholera appear than the salts were again in requisition, and the *newspapers* speedily informed us that the great Dr. Stevens was curing cholera, as no other man had cured it, by—salts. Now if reasoning is to guide us, there is no great resemblance between YELLOW FEVER and BLUE COLLAPSE, but then the blood is black in both, and salts turn blood in a porringer red, and keep the deep sea from stinking, therefore they are applicable to yellow fever and cholera, therefore they will cure yellow fever and cholera, therefore Dr. Stevens can cure yellow fever and cholera, which no one else can—quod erat demonstrandum. Such, we assure our readers, is the essence of Dr. Stevens' theoretical basis of the splendid practical discoveries, which he tells us, fond man, " will be sure to last." But we will let the Doctor talk in his own tongue. As a sample of his method of reasoning we will give the following passage from the preface.

> " But fever, at least such as is met with within the tropics, is evidently not the effect of a nervous impression; neither are those fevers produced by local inflammation, either in the brain or the stomach: for often the nervous system is not affected until after the attack; and in certain fevers of a most malignant character there is no crust on the blood; whilst in these, as in cholera, in place of inflammation, there is, in the first stage, a want of circulation—not merely in a part, but in the whole system—which is directly the reverse of inflammatory action. But every drop of the blood is deranged even before the attack, and after this, every fibre is affected, whilst the brain and all the organs are under the influence *not always of organic, but invariably of functional disease.*" xvii.

Now let us look at the Doctor's ratiocination. " Fever," says he, " is not the effect of a nervous impression," "*because* the nervous system is often not affected till after the attack," (assertion 1,)—because "every drop of blood is deranged even before attack," (assertion 2;) "and after this every fibre is affected," (assertion 3,) " whilst the brain and all the organs are under the influence not always of organic, but invariably of functional disease," a conclusion which seems to us to have as much to do with the question at issue, as with the doctrine of Rosicrucius. Again, " fever," says this learned Theban, " is not produced by local inflammation in the brain or in the stomach, for in certain fevers of a most malignant character there is no crust on the blood, whilst in these, as in cholera, in place of inflammation, there is in the first stage a want of circulation—not merely in a part, but in the whole system, which is directly the reverse of inflammatory action." The upshot of this profound piece of reasoning is merely this, that the Doctor is a humoralist of the first water; in fact he is compelled to be so, else where would be the use of his salts " that preserve the waters of the deep." The very motto on his title page is significant of his creed, and its Levitic origin a sample of his physiology—

> " For the life of the flesh is in the blood."—*Levit.* xvii.

The Doctor, we perceive, not only maintains the life of the blood, but actually places in it the life of the flesh also. The wind may be said to be the life of the mill, and on this analogy the blood may be allowed to be the life of the flesh; and as such sort of reasoning is adapted for Dr. Stevens, let us leave him in his Paradise, to fool it at his pleasure.

We do not intend to moot the issue between the Solidists and Humoralists, least of all would we contend with such a personage as Dr. Stevens. Who would seriously argue with a Doctor who writes such sentences as these—"for the vital fluid is the pabulum of the solids, but in fever it is *always diseased, and* (mark the beautiful climax of argument) to use the language of the best observer of Nature that perhaps ever lived, it often, as in cholera, 'turns to an infected jelly,' and those who neglect to attend to this can never be generally successful in their practice." This last admonition is absolutely alarming. Only think of the world having lasted some sixty centuries, whilst the rabble of surgeons and physicians have gone on practising as if they did not know that the blood may turn to an infected jelly! It is really distressing to think what a sacrifice of human life must have been occasioned by this ignorance or neglect, but then, on the other hand, it is delightful to imagine what a saving must hereafter ensue from a proper regard to it. Now that we have read this passage, we cannot exclaim with the Lydian King, that we have lost a day! No, no, the sublime idea of an 'infected jelly' shall henceforth be our beacon and watchword in every trying hour.

In order that our readers may obtain a mathematically precise idea of this stupendous work, we subjoin the following statements. It consists of—Dedication to the King of Denmark, 2 pages—Preface, 14 pages—General Observations on the Blood, 27 pages—on Animal Heat, 18 pages—on Respiration, 26 pages—on the Latent Power of Attraction, a very mysterious section, 38 pages—on Vitality of the Blood, 16 pages—on the Modus Operandi of *Agents* on the Living Body, a subject which, at some leisure hour when the cholera has ceased in Cold Bath Fields, the Doctor may compress in his fashion into as many volumes as he pleases, 28 pages—General Observations on Fever, 190 pages—Copy of a Paper which was read at the College of Physicians on the 3d of May, 1830, 28 pages—Observations and Correspondence relative to the Fever at Trinidad, in 1828, (that is, abuse of Drs. Hacket and James Johnson, "a right pleasante and conceited masque,") 61 pages—on Cholera, 68 pages—Appendix, 8 pages. The total is 523 pages, and considering the sort of stuff impressed upon them, we hereby offer eternal renown to any patient man who will undertake to swear before my Lord Mayor, or any justice of the peace, that he, the said patient man, has read the same without skipping or dozing through any quantity exceeding ten pages at a time.

We can imagine some daring spirits making the trial, and as fancy pictures their bold essay, Pope's celebrated description of the prize proposed to those who could wake over Henley's periods or Blackmore's numbers, rises in all the vivid intensity of ludicrous reality before us.

> "The clam'rous crowd is hushed with mugs of rum,
> 'Till all tuned equal, send a general hum.
> Then mount the clerks, and in one lazy tone
> Thro' the long, heavy, painful page drawl on;
> Soft creeping, words on words, the sense compose,
> At every line they stretch, they yawn, they doze.

> Who sate the nearest, by the words o'ercome,
> Slept first; the distant nodded to the hum.
> Then down are rolled the books; stretch'd o'er 'em lies
> Each gentle clerk, and mutt'ring seals his eyes.
> As what a Dutchman plumps into the lakes,
> One circle first, and then a second makes."

We will candidly confess that we have several times given up the odious task in disgust, and if at last we have contrived to get through the volume, God forbid that we should perpetrate any thing like an analysis of the verbiage and twaddle it contains. As a specimen of the knowledge and information to be derived from it, we take at random the following passage which just catches our eye on the subject of animal heat.

> "Every part of the system evidently provides its own heat; and that the quantity generated depends upon the rapidity with which the blood circulates through the extreme vessels, is very evident from what occurs during the process of inflammation. In this disease, though the respiration is not more frequent than in health, yet when the circulation is greatly increased in any one part of the body, the quantity of heat evolved in the affected part is infinitely greater than it is in the rest of the system, where the blood is circulating at its usual rate." 33.

So it is evident to Dr. Stevens that every part of the system provides its own heat. It may also be evident to him that the moon is composed of green cheese, but it does not therefore follow that all thinking persons must be sunk to his level. In point of fact, there is evidence of no such thing; nor will any of our readers fail to see the arch-absurdity of this stupid dogma. Again, says the sçavant, "when the circulation is greatly increased in any one part of the body, the quantity of heat evolved in the affected part is infinitely greater than it is in the rest of the system where the blood is circulating at its usual rate." Was there ever such arrant nonsense? How, in the name of common sense, can the *circulation* be greatly increased in one part of the body whilst the blood be circulating at its usual rate in the rest of the system? But this is of a piece with the rest of the precious production, and we really should be ashamed to criticize the views, if views they can be called, of Dr. Stevens. The utter absurdity of almost every page is only relieved by the amusing array of names dragged in, as some one said on a similar occasion, by the head and shoulders, to give an air of learning and research to the lucubrations. One is irresistibly reminded of the lines in the Dunciad—

> Next o'er his books his eyes began to roll,
> In pleasing memory of all he stole,
> How here he sipp'd, how there he plundered snug,
> And suck'd all o'er like an industrious bug.

Our author, indeed, unlike the great man thus immortalized, seems rather to take a pride in *naming* the persons he quotes, and appears to feel the same delight in talking of the Brodies and Prouts, as a retired shopkeeper in recounting the civilities of his dear Lord John, or his very kind neighbour Sir William.

As an instance of the unmeaning manner in which other persons' ideas and experiments are introduced we subjoin the opening of the chapter on respiration.

"It has been asserted, that the purification of the blood is the effect of the vital principle; but if so, then the air must possess life; for Priestley ascertained that a similar change is produced when *dead* blood is exposed to the air, even through the medium of a dead membrane; and how little either the brain or the nerves are essential to this process has been well proved by the more recent experiments of Mr. Brodie, who found that, when respiration was maintained by artificial means, the blood continued to be purified in the lungs, even for hours after the head of the animal had been cut off; and as this fact is unquestionable, we must look, not to the nerves or the living principle for an explanation of this process, but to the agency of some other power." 45.

The fine bewilderment displayed in the foregoing passage must be too apparent to need exposure. But we repeat that, turn which way we will, we are met with the same stuff, and we positively would not insult our readers by a serious notice of any more of it. We have another duty to perform, and passing over the four hundred and twenty-nine pages of nothingness that precede the chapter on cholera, we come without further hindrance to that subject. We do this the more willingly as Dr. Stevens' speculations on fever and its treatment are not worth our notice, and his statements respecting the fever at Trinidad are sufficiently exposed in the letter from Dr. Hacket in the present number of this Journal. Dr. Stevens enjoys the proud satisfaction of seeing a reviewer, with some character to lose, deliberately assert that he does not even think his views worth ridicule, whilst an able and estimable physician to a military hospital publicly contradicts his assertions. We do not envy him.

Before we pass to the consideration of the chapter on cholera, we will devote a few, and but a few lines, to Dr. Stevens' abuse of Drs. Johnson and Hacket. We shall not rip up the controversy; Dr. Hacket *has* replied, and Dr. Johnson will not. There are some persons over whom victory in a personal contest is humiliating, and such an one is Dr. Stevens. Let him rail then as he likes at Dr. Johnson and Dr. Hacket, and he may be certain that neither of these gentlemen will in future stoop to notice his abuse. Dr. Stevens is not the man to do them injury, and truth would be a scurvy protectress indeed, if her shield received a dint from the shafts of such an adversary. We therefore declare, once for all, that Dr. Stevens may write as many letters as he pleases, in as many journals as chuse to insert them, against Dr. Johnson and Dr. Hacket, without the most distant expectation of the honour of a reply. It is not with Dr. Stevens we are now contending, for, independent of our dislike of personalities, we should feel ashamed to be engaged in so mean a strife. We are demolishing his absurd and contemptible notions, and we venture to say that, after this exposure of the 'saline treatment,' it will sink into utter obscurity overwhelmed by public derision.

Dr. Stevens sets out with condemning the conduct of both the Boards of Health. He then proceeds to say that he has reason to dread that "the metropolis is at this moment slumbering on a volcano of pestilence." This, as the newsmen say, is alarming intelligence, and the metropolis will be greatly obliged to Dr. S. for waking it out of its nap. The volcano, however, is after all a mere popgun, for Dr. S. immediately informs us that "when cholera is taken in time, and *properly* treated, it is in the majority of cases, almost as easily cured as either the common typhus or the marsh fever." After proceeding, in a pitiful tone, to lament that the *facts* which he had stated, relative to the effects of salts on the blood, "appeared for a time to have been almost forgotten," a type of the utter oblivion that will speedily overtake them, he proceeds to say that some gentlemen, "who consider their profession as something more than a trade," advocated the saline treatment.

The public will mark the modesty of this gentleman. Those who do not laud his trash are denounced, by implication, as men who make their profession a trade; whilst those who may happen to approve of his notions are instantly held up as models of disinterestedness and honour. We stand up, in the name of the many professional men whom this Dr. Stevens libels, and repel his foul and foolish slander with utter scorn. If such men as Sir Astley Cooper and Dr. Prout have inadvertently patronized him, we know that they will repent their having been deluded into countenancing his *saline* absurdities. Those who have not been led away, will have the satisfaction of feeling that they have never been praised by Dr. Stevens.

Proceeding to wade through this article on cholera, we are next presented with two letters which appeared in the Medical Gazette, of which it is difficult to say which is most absurd. The first article, or letter, for we know not which it is, was penned by a Stevenite; the other is a sort of second in the duet, by the Doctor himself. The little endearments that pass between them remind one of the caresses of Mrs. Hardcastle and Tony Lumpkin. As they both consist of a sort of exposition of the Stevenian principles, and may be resolved into the Koran of this medical prophet—that salts make black blood in a porringer red, and acids make red blood black, with a number of wise corollaries thereto tacked, we need not exhaust our own patience nor our readers' by much further notice of them. We shall, therefore, only notice a passage or two illustrative of the accuracy and sagacity of this Siamese couple. For instance, No. 1 affirms—"this method of treatment, which Dr. Stevens has the merit of having first proposed, is gaining ground in the West Indies, &c. &c." The etc. etc. is certainly very significant, and a great deal is judiciously left to the imagination. Perhaps we may be permitted to transfer to vulgar prose the hidden sense imprisoned in these cabalistic symbols. We are enabled to do this by Dr. Hacket's opportune letter, in which we find the following glossary on the passage above quoted.

"I shall now finally state, in answer to all Dr. Stevens urges in his communication, and to shew the profession generally of how much value in practice his doctrines were, and are yet considered in the treatment of tropical fever, that, as far as I could learn, throughout all the following islands, which I lately visited on duty, there is not one convert to his opinions, and that the practitioners generally throughout them cry psha! both to his writings and pretensions at Antigua, Dominica, St. Lucia, Barbados, Tobago, Trinidad, Grenada, St. Vincent. For the last fifteen months that I had been stationed at Antigua, *not one particle* of his 'sodaic mixture' was administered to a *single fever case*, and yet our practice had been equally successful there as at Trinidad."*

So much for the ground that is gained in the West Indies.

---

* Vide Dr. Hacket's Letter, p. 591, Extra Limites.

Let us now look a little at the letter of Dr. Stevens himself. It begins thus.

"As I have never seen even one case of the Indian cholera, of course I can only judge of the treatment of that disease by reasoning from analogy, betwixt this and other malignant fevers which I have actually seen; but probably I was not far from the truth when I stated, that the practice which I had found so useful in the malignant fevers of the western world, *would be equally successful in the treatment of all other forms of malignant disease; and perhaps, also, after this treatment has been fairly tried, the outline of the practice in all malignant diseases will ultimately be nearly the same.*" 440.

Well done! The salines will cure all forms of malignant disease, and the outline of practice will be nearly the same in all. We shall soon have a malignant millennium, and the Procrustian tumbler of salt and water will put out all fevers of all complexions. We shall next subjoin a specimen of another kind, a sample of the Doctor's peculiar ratiocination.

"The sickness of the stomach which is so generally met with in the commencement of all those fevers that are produced by the specific aerial poisons, is probably the effect of the poison itself, which is thrown out of the circulation, and causes irritation in the gastric organs, in the same way that tartarized antimony produces nausea and vomiting when we inject a small portion of that agent into a vein: when proper remedies are used, that sickness at the stomach which begins with the disease soon passes away; but the peculiar irritation in the gastric organs which comes on at a later period, and which is often so distressing in the last stage, is evidently in these fevers produced in a great measure by an excess of acidity in the stomach. This may perhaps arise from the decomposition of the saline ingredients of the blood by the nervous or electric fluid which appears to exist in excess in all fevers, but particularly in those of a malignant character." 441.

Clever analogical reasoning this, to compare the nauseant effect of a poison, "thrown out of the circulation," with that of tartarized antimony thrown into it. Then, the sublime idea that the acid is produced by the decomposition of salts by "the nervous or electric fluid, which appears to exist in excess in all fevers." O rare physiology; O happy pathologist! Again—

"There is no question of the fact, that there is in all the malignant fevers of the new world, particularly in the last stage of these diseases, an excess of acids in the alimentary canal, which extends from the very tip of the tongue to the verge of the anus." 441.

Now does Dr. Stevens know, or does he not, that if a piece of litmus paper be applied to the tongue of any one in health, it is rendered acid—in fact, that the saliva is acid? If he does, then why should he wonder at the circumstance in fever; if he does not—but why should we express what every one must feel.

"When we apply at this period of the disease a piece of litmus paper to the foul or red irritable tongue, the test is reddened almost instantly; and when we apply the same paper to the fluids ejected from the stomach, it is reddened almost as suddenly as if it had been dipped in a pure acid." 442.

Did ever Dr. Stevens test the matters vomited, under ordinary circumstances, from the stomach? If he did, we need not tell our readers that he found it acid, at least we never saw it otherwise, and God knows we have consumed many a sheet of litmus paper in this and similar experiments.

In fact, it is lamentable to see the intelligence of the profession insulted by such trumpery arguments and ridiculous assertions. The story in the Arabian Nights' Entertainments, of the enthusiast who purchased some crockery, and arranged the mode of making his fortune and procuring the hand of the Princess of Bagdad, must be familiar enough; Dr. Stevens' saline crockery is to do as much for him.

"By this treatment we not only remove that irritation and severe burning in the stomach which is so distressing to the patient, and even so destructive to the gastric organs, but we gain another point, which is, at this period of the disease, of still more importance than the mere removal of a local irritation. The fixed acids are, as I have said, immediately neutralized by the alkali of the carbonate. The muriate of soda, and the other natural salts of the blood, are instantly formed in the stomach itself. Now we know that these salts do enter the circulation; we know also that they mix with, and become a part of the circulating blood; we know that they change its properties and remedy its morbid condition; we know also that they add to the stimulating power of the circulating current, and enable the heart to keep up its action.

In consequence of this addition of saline matter, the kidneys, and the other secreting organs, continue to perform their functions. The skin does not become yellow, nor the breath fetid; neither is the mortality one-twentieth part so great as it had been under the old modes of treatment. In fact, the successful results which have already followed the use of the above practice, prove that the saline remedies are the agents of all others the best that we yet know of, for the successful treatment of malignant diseases." 444.

Like a lively flea, the Doctor hops with surprising vigour from assertion to assertion, until he satisfactorily ascertains that his treatment is the very best that has ever been devised by the wit of man. Be it so.

"The dark colour of the blood, which we observe in the beginning of pestilential fevers, is the effect of the poison on the vital fluid; but the blackness in the last stage of these diseases is produced *by the loss of the saline ingredients*, which I can prove are beyond all question the true cause of the red colour of healthy blood." 445.

In this short passage there are three assertions, not one of which is easily proved. But this is of a piece with all the rest. We solemnly declare that we do not believe there has ever been published a medical work containing a greater mass of reckless, unproved, and improbable assumptions, stuck together with such a putty of ridiculous reasoning. A connected criticism of such twaddle would be next to impossible, for one would have to argue every sentence. The severest censure is to expose passages, in the nakedness of their nonsense, to public laughter. We are told by Stevens, that the salines should be given in the collapse stage of fevers or cholera, because they enter the circulation and are stimulants; but such is their wondrous power, that we are also to give them in the stage of reaction, because they prevent a black and dissolved state of blood.

"When this (fever) is effected (by the stimulant salines), should the re-action run high, the excitement may be reduced by the use of the lancet, and the typhoid symptoms, which sometimes afterwards occur, may probably be prevented by the subsequent use of the carbonate of soda and other saline medicines, which we know do possess the power of preventing that black and dissolved state of the blood, which is, in reality, in fever, the true cause of the nervous as well as the other bad symptoms." 449.

Thus we are told the usual story; the remedy is good for the cold stage and good for the hot—cures yellow fever and blue cholera—is the best treatment known for *all* malignant diseases. We suppose that by and bye Dr. S. will denounce all remedies but salines, like the Kaliph Omar, who burnt the Ptolemæan Library because it contained more or less than the Koran.* Let us take another specimen of Dr. Stevens' reasoning.

"I know it will be asked, why have the citric and other acids been successful in scurvy, where the blood is darker than it is in health? To this it may be answered, that the scurvy is not, like the cholera, or the yellow fever, a disease that causes death in a few hours, or a few days; and therefore medicines that may be used without causing immediate death in the one, cannot be used in the others with equal impunity. My own conviction is, however, that there is no one disease in the whole catalogue in which the profession has been so much misled as in the very disease now under consideration. During a residence of twenty years in the West Indies, I have only seen one case of scurvy, and *that case was decidedly brought on by the excessive use of citric acid, which an American gentleman had been recommended to use as a preventive against the yellow fever*. His own conviction, as well as mine, was, that the scorbutic symptoms had been brought on by the acid. This was immediately laid aside, and, under the use of the carbonate of soda, he was completely cured in three weeks." 451.

Thus, Dr. Stevens never saw in the West Indies but one case of scurvy, and on the strength of this he denies the efficacy of the vegetable acids, a fact established by facts of a very different description from those of Dr. Stevens'. It would be very inconvenient, in sooth, to allow that a disease, characterized by black and dissolved blood, can be brought on by a diet containing much of the identical salt which is to remove such a condition. See to what shifts the Doctor is reduced, when, to answer such an objection, he tells us that one gentleman made himself ill by "an excessive use of citric acid," and was cured by carbonate of soda—a very likely circumstance. The quibble on scurvy not being, like yellow fever, an acute disease, is laughable enough, and would be apparent to a school-boy.

We now arrive at the Cold-Bath-Fields affair. We have hitherto had to do with doctrines too absurd to require argument, assertions manifestly unsupported or insupportable, and facts which, however one might disbelieve, it would be difficult and troublesome to disprove. But here we have the practical application of the saline treatment—we have the doughty Doctor dispensing its benefits with his own discriminating hand. All this happens in our own country, in some degree beneath our own eyes; and, therefore, we have a better opportunity of testing the value of the practice than when the scene of action is in the Danish West India Islands. To this ordeal Dr. Stevens should confidently appeal, if his cause were good. We shall see how he will come out from it. As it is of consequence to lay the whole affair before our readers, we shall do so most impartially. Dr. Stevens may be assured that, from us, he shall have justice, if we do not grant him mercy.

"In the beginning of April, I received a note from Mr. Pout, a medical gen-

---

* On the gates of this library were inscribed the words ΨΥΧΗΣ ΙΑΤΡΕΙΟΝ—Physic of the Soul, whence Pope's lines—

"From shelves to shelves see greedy Vulcan roll,
And lick up all their physic of the soul."

tleman in Albany-street, who called to inform me that the cholera had broken out in the prison at Cold Bath Fields, and that he had been requested by Mr. Wakefield, the surgeon who had charge of the prison, to say that he would be glad to show me the cases; and from what he had heard of the saline treatment, he should be very willing to give it a trial—the more so, as he had now no longer any faith in the common remedies.

On the receipt of this message, I immediately went to the prison; and after some conversation with Mr. Wakefield on the subject, he not only agreed to adopt the saline treatment, but invited me to attend the cases along with him. He consented also that Mr. Crooke, a young medical gentleman who had lived with me for several years in the West Indies, should be allowed to remain constantly in the prison, to see that the medicines were faithfully administered, *as well as to take notes of the cases.*" 458.

Here was every thing Dr. S. could wish—a *prison* to himself—a surgeon evidently anxious to second him—and a pupil of his own, Mr. Crooke (a rather ominous name) to take notes. What else could be desired? The following is the outline of the treatment pursued by Dr. Stevens "not only in the prison, but every where else." To prevent a chance of even misconception, we shall give his own words.

"First. The treatment was generally commenced with a Seidlitz powder, which was given with a view of lessening the gastric irritation, and partly for the purpose of removing the diseased secretions from the intestinal canal.

Secondly. When the stomach was irritable (which it generally was) a large sinapism was immediately applied to the epigastric region, and where the patients were cramped in the extremities, frictions were used with hot flannel. The pain produced by the spasms in the muscles were not only relieved by the frictions, but by this and the application of sinapisms to various parts of the body, the quantity of animal heat was increased, and this, I need scarcely say, is an object of great importance in the treatment of cholera.

Thirdly. A powder containing—

Carbonate of Soda, ʒss.

Muriate of Soda, ℈j.

Chlorate of Potass, grs. vij.

was dissolved in half a tumbler of water, and given soon after the Seidlitz. In severe cases, the above powder was administered every half hour. In those that were less severe, it was used every hour, and in some malignant cases it was given every fifteen minutes. In short, it was given more or less frequently according to the circumstances of the case, and continued until the circulation was fairly restored; it was then given at longer intervals, and when the re-action was completely established, it was left off by degrees.

Fourthly. Where the stomach was irritable, the use of the above powder was occasionally suspended, and common effervescing mixtures, or small doses of the common soda powders, with an excess of the carbonates, were frequently used, until the irritation was lessened, and then the carbonate of soda with larger doses of the chlorate of potass were generally given without the addition of the muriate of soda, and frequently in such cases the chlorate of potass was given by itself, in doses containing ten grains each.

Fifthly. A solution of muriate of soda was also thrown up into the intestines, at as high a temperature as the patients could well bear this saline fluid.

Sixthly. In two very severe cases, which occurred out of the prison, the patients were put into a hot saline bath with evident advantage. It is well known, that a hot saline fluid is a better conductor of heat than fresh water at the same temperature; but, independent of this, a part of the saline ingredients may be absorbed from the skin, and the patients may also be benefited by respiring the hot saline vapour. It is but fair to state, however, that this means, which was evidently beneficial in the cases in which it was tried, was proposed by Mr. Marsden, one of the surgeons to the Free Hospital in Greville-street.

Seventhly. Seltzer water was allowed *ad libitum*, when the patients expressed a desire for something to drink. A strong infusion of green tea was also occasionally used, in severe cases, apparently with advantage.

Eighthly. It was considered essentially necessary to keep a large fire, both night and day, in every room where there was a patient with cholera. It is now well known that in by far the majority of cases, the collapse commences betwixt two o'clock in the morning and six, a. m., or, in other words, at the period of the twenty-four hours when the atmosphere is coldest: from which it appears that external cold acts as an exciting cause to the state of asphyxia. But independent of this, we have seen that the degree of force, with which oxygen can remove carbonic acid through the medium of a membrane, depends, in a great degree, on the temperature of the two fluids. Now, when the temperature of the blood is so very low, as it is during the state of collapse, and if the air which the patients then breathe be also cold, the small quantity of carbonic acid which exists in the black venous blood, will not be attracted by the cold air, and consequently this of itself may be one cause of the sudden death.

Ninthly. It is necessary to be very careful not to dismiss the patients as cured until they have been, at least, several days completely out of danger. Two of the cases which proved fatal in the prison, at Cold Bath Fields, were lost from our not having been at that time sufficiently aware of the importance of this.

Tenthly. The patients ought not to be allowed to use one particle of solid or indigestible food, for at least five days after they have recovered from the state of collapse. We nearly lost more cases than one, from the too early use of solid indigestible food; and one woman, a nurse in the London Free Hospital in Greville-street, actually died from this cause, after having been considered as completely out of danger from a most violent attack of cholera accompanied with collapse.

Eleventhly. Those who put their patients under the saline treatment, ought to trust almost entirely to this; for if they use calomel, brandy, or other destructive agents at the same time, they will do little good; but above all, *not one particle of opium ought to be given internally;* for, from what I have seen, I consider this to be as fatal in cholera, as it is in the last stage of either the African typhus or the seasoning fever of the West Indies. Where the stomach, however, is extremely irritable, *about twenty-five or thirty drops of laudanum, diffused in a little tepid water, may be injected with a small syringe into the rectum, not only with impunity but considerable advantage.*

When the stomach is very irritable, small quantities of milk, with carbonate of soda, may be given occasionally; and when we use the saline powders in such cases, they ought to be dissolved in a very small portion of water.

When the case is exceedingly malignant, or where we are called in late in the disease, and find the patient already in collapse, we ought then to have recourse to the most active measures. An ounce of the muriate of soda, with half a drachm of the chlorate, or the muriate of potass, should be given immediately in cold water, and repeated, if necessary, every half hour, until the patient has taken about three doses of this strong solution. *Should re-action be brought on by this,* it may then be kept up by the common saline powders; but should this experiment fail, we may then, as a last resource, give the patient another chance for life, by injecting a saline fluid into the veins.

The ejections, and every other source of impurity, ought to be immediately removed from the room where the patients are; and the infected wards should be fumigated at least twice a day with gunpowder, and every particle of suspicious clothing, bedding, &c. should be boiled, for at least half an hour, in a strong solution of common soda." 463.

We shall only make two observations on this. If opium be so pernicious why should Dr. S. find benefit from an enema of laudanum? Is there any power or property in one end of the intestinal tube of correcting an agent which is noxious when introduced into the other? Is not this so far from being true, that some practitioners, the Baron Dupuytren, for example, believe that opium exerts its specific effects most powerfully when used in the form of injection? In fact, this passage is a pathological shuffle. The Doctor found that his bumpers of salt and water would not arrest sickness, and, being compelled to give opium, he compounded with his creed, and gave it by the anus. The other observation we have to make is this. The Doctor applies his saline treatment to clothes as well as Christians, the common soda neutralizing, we suppose, the *acid* cholera that lurks in the dirty linen. What shall we say to the explosions of gunpowder in the wards at least twice a day. We wonder the Doctor did not borrow a park of artillery; these periodical fire-works must certainly have relieved the ennui of Mr. Crooke, and formed an agreeable divertisement amidst the monotonous uniformity of diarrhœa. Having given the treatment, the Doctor next offers "what he believes is a fair statement of the outline of the result."

"The three first cases which occurred in the prison were treated by Mr. Wakefield in the common way—with opium, brandy, the hot-air bath, &c.; but they all died after a very short illness. Almost immediately after this, another case was treated in a similar way by another practitioner, who had been sent for to the prison during the night, in consequence of Mr. Wakefield being unwell at the time. This gentleman was not then aware that any new practice had been adopted in the prison. He treated the patient *secundum artem*, with brandy, opium, and chalk; but the result was, that this patient was past all hopes of recovery before either Mr. Wakefield or myself saw him in the morning;—consequently, in the four cases that were treated in the prison in that way, there were four deaths and not one recovery under the common practice.

It may be proper to state, that previous to the beginning of April there were no bowel complaints in the prison, and the whole of the prisoners were then as healthy as they generally are at that season of the year. The first case that was reported to the Board of Health occurred on the 5th of April;—the saline treatment was commenced on the 8th. There were in all at that period about one thousand three hundred souls in the prison; and from the 8th of April to the cessation of the first epidemic, there were at least one hundred individuals who were evidently, *more or less*, under the influence of the poison.

In about fifty of the above cases, the patients were attacked with *a bowel complaint*, and most of them had, *more or less*, irritation at the stomach. The fluids that were ejected were generally deficient in bile; and the bowel complaint was attended with the following peculiarities:—

First. The inclination to go to the night-chair came on more suddenly than it generally does in cases of common diarrhœa.

Secondly. The ejections were less bilious than in common diarrhœa; and opium, chalk, astringents, &c., which are generally useful in cases of common bowel complaints, *were of no use in checking the diarrhœa, which occurs when the patients are under the influence of the cholera poison.* These remedies were chiefly used in cases which occurred out of the prison; but they evidently had no effect in checking the specific ejections which are produced by the cholera poison; and this I presume was the cause of the diarrhœa which occurred in the fifty cases in the prison to which I refer. The whole of these were immediately put under the saline treatment, and this appeared to give an immediate check to the disease; and I believe it was owing to the saline remedies, *as well as to the*

*circumstance of their being constantly kept in a warm room, and well taken care of,* that not one of these cases ended in collapse: consequently, though they were constantly breathing in an atmosphere completely impregnated with the poison, yet not one of them was lost.

There were also about thirty-one similar cases, in which the above symptoms were still more distinctly marked, and *in many of them the bowel complaint was more or less accompanied with cramps.* These were all treated in the same way with the non-purgative salts, and in three, four, or five days, every one of them were sent from the observation ward, as we believed at the time, completely out of danger. I am sorry, however, to be obliged to add, that two of these cases which had been unfortunately dismissed too soon, and sent back as cured to the cold wards of the prison, were attacked with collapse during the night; and *before they could again be put under the saline treatment,* their stomachs were so irritable that they could scarcely retain even a teaspoonful of water; and both these cases proved fatal in a very short period from the commencement of the collapse.

In addition to the eighty-one individuals already referred to, we had about nineteen cases in the prison, where the patients were either attacked with the disease, and got into a state of asphyxia in the cold wards of the prison during the night, or where the stomach was so irritable in the first stage *that it could not retain the stronger salts.* In almost every one of those cases the disease assumed a most malignant character. These were all treated with the energetic non-purgative saline remedies; and in the nineteen malignant cases to which I now refer, we had eighteen recoveries, and only one death: consequently, the total number of patients, who were all evidently under the influence of the cholera poison, was about one hundred, yet in those cases where we trusted almost entirely to the saline practice, we had only three deaths, and ninety-seven recoveries.

In corroboration of the above statement, I will insert here the following letter from Mr. Wakefield, which was published in the Medical Gazette for April 28, 1832." 466.

According to Dr. Stevens' own shewing, fifty individuals out of about one hundred, had "a bowel complaint, and most of them had more or less irritation at the stomach." Of these none died. Now Dr. Stevens asserts that the remedies which are generally useful in common bowel complaints are of no use in checking the diarrhœa which occurs when the patients are under the influence of the cholera poison, thereby of course implying that of the fifty persons so attacked with diarrhœa many would be lost under ordinary treatment. We contradict Dr. Stevens. If by diarrhœa arising from the influence of the cholera poison, Dr. Stevens means that diarrhœa which has lately been so prevalent, and which, when neglected, has run into absolute and fatal collapse, we most positively deny that "common remedies" will not cure it. We need not appeal to our professional brethren in confirmation of our statement, as the remark has been publickly and frequently made since the prevalence of cholera in England. It must be remembered too, that the Cold Bath Fields patients were immediately attended to, "constantly kept in a warm room, and well taken care of." Where is the wonder that out of fifty such cases of diarrhœa none were fatal. We are not even surprised at the salt and water having done no mischief.

But there is one statement so extraordinary that we cannot but take notice of it. Two patients sent back to the cold wards of the prison, "were attacked with collapse during the night, and before they could be again put under the saline treatment their stomachs were so irritable that they could scarcely retain a teaspoonful of water; and both these cases proved fatal." What then, does it occupy some time to put patients upon the saline treatment, and will this be of no avail if there is great irritability of stomach? One would think that the salt and water could be soon procured in a place where it must have formed a staple commodity—in a place attended by two saline doctors, and inhabited by a saline house-surgeon! Yet it was not so. The saline treatment, we infer, will not do when there is extreme irritability of stomach. Now, such is our simplicity, that we always considered this symptom as one attending a large proportion of the cases of malignant cholera. We have seen this instant a case which will probably be fatal, where this is a very prominent symptom. Yet it seems that salines did not succeed in two instances, because this was established! Can none of our readers smell a rat—can they not perceive that two severe cases having really occurred, the salines were of no more use than ditch water?

Eighty-one cases then out of the hundred were of the kind alluded to—cases which would never have been fatal under ordinary care and treatment. The remaining 19 patients "were either attacked with the disease and got into a state of asphyxia during the night, or the stomach was so irritable that *it could not retain the stronger salts.* Of these 19 one died.

Next we have a letter from Mr. Wakefield, crying double to Dr. Stevens' just quoted. There are only two additions requiring any notice. In alluding to the 81 cases before referred to, Mr. Wakefield goes even farther than Dr. Stevens—he *believes* that one half would have been lost under ordinary treatment!

"I believe also, that had they either been left to themselves or improperly treated, the majority of these cases would have run into a state of collapse, perhaps in a few hours; indeed I have little doubt that the one-half of them would have been lost under the practice which is generally adopted in the treatment of this disease." 468.

Agreeing with the doctrine that no man can be justly blamed for his beliefs, as they are in a great measure independent of his *inclination,* we only record Mr. Wakefield's, and pass on. The second point is this. Mr. W. states that he has had one most malignant case in the New Prison at Clerkenwell, where the patient was in a complete state of collapse before he saw him.

"His extremities were cold; his pulse at the wrist was entirely gone; he had the cholera voice, and his tongue was icy cold. This man, like those in the other prison was immediately put under the saline treatment with the happiest effects, and I consider him now in a state of convalescence." 469.

Many such cases as these would be important, if true. Such was the first irruption of cholera which occurred in Cold Bath Fields Prison. It commenced in the beginning and terminated on the 30th of April. The second irruption began on the 3d of June.

"In this instance it commenced amongst the females, and soon spread almost all over the whole establishment, and is now at this moment much more virulent, and I am sorry to add, *more fatal,* than it has ever been at any former period. In the first case that occurred the woman was attacked on the night of the 3d, and died on the 5th. Her sister, who attended her, was next taken ill, but recovered under the saline treatment.

Soon after the commencement of this second irruption, I called at the prison, and there were then four cases. These were under the saline treatment, and as they were all doing well, I did not return. On the 21st of June, however, I received a note from Mr. Wakefield, requesting me to meet him at the prison as soon as possible. When I went there, I found about twenty patients with cholera, and out of this number five were actually dying. *There was one obvious cause for this, which I do not feel myself at liberty to point out,—suffice it to say, that it originated from either a mistake or neglect on the part of the nurses who administered the medicines.*

A saline fluid, similar to that which had been used at Leith, was injected in two cases, into the veins; but the one died almost immediately, and the other though he rallied for a time yet he also ultimately died.

From the commencement of this second irruption there have been in all, about eighty-one cases: *many of these* have been of the most malignant description. Out of this number *there have been thirteen deaths*, and the other sixty-eight have either recovered or are now apparently nearly out of danger; but new cases are brought into the infirmary almost every hour. They are all of them however now under the most energetic treatment, and I sincerely trust that the mortality of the disease will be arrested in its progress." 472.

In another place Dr. Stevens adds—"We have now had, during the present irruption in the prison, one hundred and five cases, and fifteen deaths, and there are at this moment in that establishment twenty-two patients *who have recently recovered from a state of complete collapse.* But though the proofs in favour of the saline treatment were as numerous as the sands on the sea-shore, still there are individuals who will deny its utility—and that too in a manner which can neither do credit to themselves nor good to humanity."

In this second irruption the cases were more severe, and what was the result? Out of 81 patients 13 died, or one in six. Now these 81, comprising all attacked, were in all probability a mixture of cases of different degrees of severity, for all practitioners must have found, that of a certain number of cases occurring in any locality the number of those decidedly malignant would not be very great. In the present instance, even Dr. Stevens, he who pretends that the cholera diarrhœa is not curable by ordinary remedies, allows that of the 81 only "many" were of the most malignant kind. Now the proportion of deaths in the malignant cases in Great Britain has been little more than one in three, yet this so vaunted saline treatment saves one in six out of 81 cases, of which only "many" were most malignant. Is it possible that this transparent veil of fallacy can obscure the truth. Again, we see by the note to the passage quoted, that the muriate of soda is too apt to run off by the bowels. This note seems to refer to saline injections into the veins, but if it be true of them, it is still more likely to be so of muriate of soda swallowed. So the saline doctrine turns out to be Hahnemannian, curing diarrhœa by producing it—a rare discovery. The doctrine however is not so contemptible, as appears by the following testimonial awarded by the magistrates of the county to Stevens, Wakefield, and Crooke.*

*(Continued at page 422.)*

---

* This article having extended to a greater length than was anticipated, it has been necessary to remove another, in order to make room for it. This will explain the erroneous notation of the succeeding articles.—*Ed.*

### XI.

### DR. STEVENS ON THE BLOOD.

*Continued from page* 336.

"At the last meeting of the magistrates of the county of Middlesex, it was unanimously agreed, that a vote of thanks, and a piece of plate of the value of one hundred sovereigns, should be presented to Dr. Stevens, for his attention to the sick, and the great success which had attended the saline treatment, in the prison of Cold Bath Fields. Another piece of plate, of the value of fifty sovereigns, is also to be given to Mr. Wakefield, surgeon to the prison; and another of the value of twenty-five sovereigns, to Mr. Crooke, a young gentleman who lived for several years with Dr. Stevens in the West Indies, and is now a student of medicine in the London University. Small sums have also been awarded to several of the nurses; and some of the prisoners, who rendered themselves useful by their attendance on the sick, are to be recommended for pardon, through the magistrates, by the Secretary of State."—*Med. Gaz. June* 2, 1832.

Orpheus, whose fiddle moved the trees and stones, was after all but a bungler when compared with Dr. Stevens. HE has moved Sir Peter Laurie.

Dr. Stevens proceeds to mention the success attending the saline treatment of Mr. Whitmore and Mr. Marsden. As, however, we consider the institution immediately under Dr. Stevens' own care, as affording the best means of testing his own practice, we shall leave those gentlemen without further notice. We have brought before our readers the whole of the Cold Bath Fields case as stated by Dr. Stevens himself, and we have shewn that, even reasoning on *his own* statements, the result is rather against his practice than for it. But this is the most favourable side of the question.

The Board of Health being constantly dinned, no doubt, with the success of Dr. Stevens, determined, very properly, to investigate his statements. The result must have been distressing to the feelings of the saline party.

In the Lancet for July 14th, 1832, there is a communication from Sir David Barry, stating the results of a visit which he made to the Coldbath-Fields Prison, on the 27th and 28th of June.

" On the night of the 25th of June ult., in a casual conversation with Dr. William Stevens at the College of Physicians, I learned, with no small astonishment, that he had seen upwards of forty cases of cholera in Coldbath-Fields Prison within the preceding 24 hours. Struck with this formidable announcement, I requested permission to see these cases with the Doctor next morning, but could not obtain an appointment with him earlier than for the 27th.

On that day I proceeded to the prison rather in a private than official capacity, accompanied by Dr. O'Shaughnessy, whose 'Report on the Chemical Pathology of Cholera' entitles him to such high consideration in everything connected with what has been lately denominated the saline treatment of that disease.

Dr. Stevens conducted us round all the wards appropriate to cholera patients. On leaving the prison, at about half-past two o'clock, p. m., I observed to him, in presence of Dr. O'Shaughnessy, that I had seen no case of cholera in the prison that day—meaning, as Dr. Stevens appeared to allow at the time, that I had seen none actually labouring under the characteristic symptoms of the disease." 455.

On the morning of the 28th a note was addressed by the Privy Council to the governor of the prison, requesting to be informed whether the statements in the newspapers respecting the frightful extent of cholera in the prison were correct. To this the governor replied that unfortunately much of the newspaper statement *was* correct, the number of persons then labouring under the disease in its various degrees being about seventy. In consequence of this deplorable insalubrity Sir David Barry was officially desired to visit the prison, which he did on the 28th inst. It will be recollected that his previous visit with Dr. O'Shaughnessy, on the 27th, was a non-official one, and it will also be remembered that at that non-official inspection of the 27th he declared to Dr. Stevens that he had not seen one case of cholera. There must indeed then have been grounds for serious alarm, when Sir David Barry finds no case of cholera on the 27th, and the governor of the prison, acting no doubt on information derived from the medical attendants, declares on the 28th that there are seventy. When pestilence rears its head with so appalling a front, it well becomes His Majesty's Government to institute an official investigation. The volcano, we suppose, alluded to by Dr. Stevens, had begun to discharge in the night of the 27th.

The minutes of the official visit shall be given in Sir David Barry's words.

" Coldbath-Fields Prison, June 28th, 1832.

" *Notes.*—Visited the wards appropriated to cholera patients in this establishment at half-past four o'clock, accompanied by Deputy Inspector General of Hospitals Maling, and Staff-Surgeon Macann, conducted by the governor of the prison, and two visiting magistrates.

Saw all the wards in which persons said to be labouring under cholera were treated, and examined individually all those said to be on the sick list, then present.

1*st Ward visited.*—Nine patients. One man who had been four days under the saline treatment for premonitory symptoms had been attacked this morning, after having been discharged from hospital. A genuine case. Attempts were making, *by a young man of colour,* to introduce the tube for saline injection into one of the veins at the bend of the arm, under the direction of Mr. Wakefield. *Tube could not be introduced, as I learned afterwards.* Fluttering pulse, livid and sunk countenance. This case will most probably prove fatal. Another man in this ward, looking thin, pale, depressed, hollow eyes, but good pulse, is under saline treatment. *When I saw him about half an hour afterwards, his tongue was cold, with a weak slow pulse.* Ward small for the number of beds; *close, hot, and oppressive, with a very large fire.*

*2d Ward visited.—Eighteen* persons *said* to be on the sick list in this ward. *Two only present, boys, apparently well.* This ward consists of two rooms; the inner a narrow slip. The *sixteen* not present were said to be *out walking.*

*3d Ward called ' No. 5.'*—Six patients on the *Cholera Hospital Book.* Five present. *One man complains of constipation of the bowels. One boy has had pain in his side and head, now better; no vomiting or purging. No appearance of cholera in the others at present.*

4*th Ward visited.*—Eleven patients. All present. One boy with slow pulse, and depression of look and spirits; may have an attack in the course of the night. *All the others looking well, with no appearance of disease of any kind.* Informed by Mr. Wakefield, the surgeon of the establishment, that the diet of the cholera patients consists of arrow-root, tapioca, beef-tea, coffee, and Seltzer-water for drink, *ad libitum,* a wine-glassful at a time.

*Convalescent Ward.*—Fourteen patients. All looking well.

*Female Ward, No.* 1.—Nine patients—all looking well. One young woman, apparently *simulating cholera;* warm skin, good pulse and tongue. Said to be a very troublesome perverse character.

*Female Ward, No.* 2.—Nine patients. One now in mild fever. Said to have been a severe case of cholera. *One young woman with bad toothach.*

The two men in No. 1, already mentioned, are the only cases which I saw with the appearance of cholera. Yet the governor assured me repeatedly, that he had shown me *all* the persons considered by the medical gentlemen as labouring under any stage of the disease, and referred to in his letter of this day to Mr. Bathurst.

The utmost cleanliness, regularity, and discipline, appear to prevail in every part of the prison, as far as I was able to judge; and the visiting magistrates, who went round the wards with us, seemed to be actuated by the most humane feelings, and to devote much time and attention to the health and comfort of the prisoners.

No new case admitted this day. (Signed) D. BARRY.
JOHN MALING.
F. MACANN.

It is almost needless to observe, that no part of the preceding notes or statements is meant to refer to any time or circumstances connected with the patients, anterior or posterior to the moments at which they were seen by Dr. O'Shaughnessy, Mr. Maling, Dr. Macann, and myself. D. B."

Now we appeal to the profession whether there was not indeed a necessity for instituting a full inquiry. On the 26th Sir David Barry is told by Dr. Stevens that he had seen forty cases of cholera in the previous 24 hours. On the 27th he visits the warehouse, and finds *not one*. On the 28th the Government is informed that there are seventy cases in the same establishment; on that very day Sir David Barry goes and finds just two. Out of 18 cholera patients in one ward (18 of those who, Dr. Stevens has formerly said, could not be cured by ordinary remedies, and one half of whom should, according to Mr. Wakefield, die) 16, proh nefas! are taking a walk. Out of six in another ward one man is constipated, one boy has pain in his head, three have no appearance of cholera, and the other is not to be seen! Out of 11 in another ward, 10 look well and have no disease of any description. In a female ward, we have nine patients, all looking well, plump, and hearty, no doubt fattening on the pestilence. Out of nine in another ward one has mild fever, and one has, *risum teneatis?* a tooth-ache. Can we wonder that Dr. Stevens, Mr. Wakefield, and Mr. Crooke obtained from the magistrates that better reward than empty praise, the solid pudding of three pieces of plate. Must not every humane and philanthropic mind rejoice that a pestilence so fearful, a visitation so awful, should have been, under Dr. Stevens and the magistrates, the means of liberating from durance vile a few deserving persons, whom the hardship of the laws and the vicious injustice of society had immured? Out of evil indeed cometh good.

Perhaps it may be said that, at the time of Sir David Barry's official visit the disease was on the decline; that the persons who were walking, talking, suffering from tooth-ache, constipation, or nothing, were, in point of fact, triumphant living witnesses to the might and majesty of the saline treatment. But this will not do; for, on the very morning of that visit the governor of the prison writes to the Privy Council to state that "*unfortunately* much of the statement from the *Globe* newspaper is correct," and that "the number of persons *now labouring* under the disease, in its various degrees, is about seventy." From this it is evident—that the Governor, acting of course on information derived, in one way or other, from the medical attendants, considered the pestilence as *then* prevailing, and the epithet

" unfortunately" must *unfortunately* convince every rational person that it was by him considered in a serious light, probably as serious as at any preceding period.

But this is not the whole of this extraordinary farce. The magistrates who had *plated* Stevens, Wakefield, and Crooke, finding that Sir David Barry's statement made them cut a figure bordering on the ridiculous, determined to put down by the weight of their wigs the saucy impertinence of a few doctors. Accordingly these magistrates held a meeting on the 7th of July, for a report of the absurdities at which we must refer to the newspapers of the day. Sir Peter Lawrie, " a name to science ever dear," especially patronized Dr. Stevens, and dubbed him a second Jenner, an exterminator of pestilence, a missionary from Heaven for the protection of man. To all this we have no objection, as Dr. Stevens possesses as good a right to the receipt of these various orders, as Sir Peter Lawrie has to the conferring of them. Another of the magistrates, Mr. Rotch, made a more specific statement, which can therefore be met by a specific answer. In alluding to the official visit on the 28th June, he asserted that it was *superficially* conducted, and much incredulity was manifested by Sir David Barry. Now our readers will remember that the visit of the 28th was Sir David's second, his first having been on the 27th in company with Dr. O'Shaughnessy. Fortunately, notes of this visit were preserved, and were published immediately on the notification of the magisterial charge. In the Lancet of July 21st, there is the following letter from Sir David Barry; it is the minute of the before-mentioned visit on the 27th inst. and serves to complete the annihilation of the saline humbug.

" June 27th, 1832, visited the Coldbath-fields prison this day, at two o'clock, accompanied by Dr. O'Shaughnessy. Learned from the Governor in his office, that the population of the prison at this moment is about eleven hundred of both sexes, including twenty-one children, that the daily discharges and admissions are about thirty-five on the average; the whole composed of malefactors, vagrants, and paupers; that the daily allowance of food to an adult is (since the introduction of cholera) 1¼lbs. of bread, 1 pint of gruel, 1 pint of ox-head soup, 6 ounces of boiled meat, four times a week, free of bone, and weighed after having been boiled. No soup on meat days.

That the deaths during the spring, summer, and autumn months last year, were only two; during the whole of last year, fifteen. That since the 3d June, there had been twelve deaths, all from cholera. That the present state of cholera cases in the prison is as reported to him, the Governor,

Men, 55 cases. Women, 15 cases.

That he had not lately seen them; he could be of no service, *and was recommended not to go into the cholera wards.**

Walked with the Governor, Dr. Stevens, and Dr. O'Shaughnessy, round the gardens and other open spaces within the walls, and recommended the prisoners' rooms to be thinned by encamping a part of the inmates in the gardens.

Dr. Stevens, with his two assistants, then conducted Dr. O'Shaughnessy and myself round his cholera wards, in which there might have been thirty or forty patients, *but not one in the collapsed stage, though thirteen cholera cases are stated to have been admitted this morning.*

There certainly was *not a single case* which from any symptoms witnessed by

---

* " The Governor accompanied me round the sick wards on the 28th, but not on the 27th June."

me *I could point out as a case of cholera.* I afterwards made the same observation to Dr. Stevens in the presence of the Governor and Dr. O'Shaughnessy. I repeatedly begged Dr. Stevens to show a recent case, a case of confirmed cholera —a case of collapse. He replied it was *unlucky* I had come that day,* and there was no case of collapse then in the house, and remarked that by his treatment, *he prevents their ever coming to the collapsed stage.* Yet twelve have died since the 3rd, two of whom died the day before yesterday; and he (Dr. Stevens) allows that several became collapsed after having been admitted into the hospital of the prison, *consequently while under the saline treatment.*

One boy admitted this morning, in bed, *has had neither vomiting nor purging*, and presents a steady pulse.

Two persons looked pale, sallow, and with sunken eyes, as if they had suffered some severe evacuations, but the pulse in both is good. Seltzer water is given as common drink.

Recommended grated openings to be constructed in the walls of the small yards and rooms on a level with the floors. The air in these places being more or less stagnant to a height of eight feet at least.

Dr. Stevens, during our visit to the prison, and afterwards on the way to the Regent's Park, repeatedly asserted, that two-thirds of the patients which he showed us, *would* have fallen into collapse, and *would* have most certainly died if subjected to the ordinary plans of treatment by calomel, opium, stimulants, &c. In short, that the present usual mode of treating this disease *is absolutely poisonous.* When asked if he had ever seen even one case of blue collapsed cholera saved, that had been treated by the twenty-grain doses of culinary salt, and by nothing else, he candidly admitted that he never had, for that he recommended, in addition to the salt, *large mustard poultices, hot* saline and *opiate enemata*, and *hot* salt and *water-baths, friction*, &c.

The substance of the above notes was written in the prison, or immediately after returning from it, and in their present state were read by Dr. O'Shaughnessy, previously to the publication of Stevens' work on the blood.

D. BARRY, M.D.

---

I have read the above notes, which I saw several days since, and have to state, that they are accurate in every respect.

16th July, 1832. (Signed) W. B. O'SHAUGHNESSY, M.D."

It was indeed "unlucky" that Sir David Barry went—unlucky for salines —unlucky for the Doctors—unlucky for the renown in futuro, and éclat in præsente, which the firm in Coldbath-Fields fondly hoped to acquire.

We shall not pursue this ludicrous, or rather melancholy subject any further—we shall not trust ourselves with the expression of what we think or what we feel. We turn from the whole affair with mingled sensations of pity and disgust, and the best that can be hoped for the "saline treatment" and its authors is, that both may be speedily forgotten.

We have cautiously abstained from discussing the question of saline injections into the veins in the treatment of cholera. We certainly do not anticipate any thing from this plan, and we feel convinced, from what we have seen and read, that it will ultimately be given up. But this stands on a very different bottom from Dr. Stevens's saline treatment—it is advocated by a very different description of persons, and supported by a very different kind of facts.

It may seem to many of our readers that, in reviewing Dr. Stevens' book,

---

* "I had come, by appointment, with the Dr. himself, made on the 25th at the College of Physicians."—D. B.

we have adopted a more severe and menacing tone than is our usual practice. We acknowledge the fact. It is neither our habit nor our inclination to be severe—we feel no pleasure at others' pain—we cannot contemplate with gratification even the writhings of a worm. But there are occasions on which leniency is criminal, and such an one we have deemed the present. Dr. Stevens will in future understand that forbearance is not always weakness, and that because a reviewer does not often use the lash, he is therefore inexpert in its employment. We know that although ourselves may be insignificant we wield a mighty power, and we trust that we entertain a due sense of our responsibility. The press is almost omnipotent for good or for evil, and Dr. Stevens might as well dream of contending with the Atlantic, as with Justice and the press together. We take our leave of Dr. Stevens, in all probability for ever.

